# Predicting outcomes within an innovative post-acute rehabilitation model for older adults

**DOI:** 10.1186/s12877-019-1147-6

**Published:** 2019-05-27

**Authors:** William B. Dieter, John P. Collins, Andrew A. Guccione

**Affiliations:** 1 Fox Rehabilitation, Cherry Hill, NJ USA; 2 Department of Rehabilitation Science, George Mason University, Fairfax, VA USA

**Keywords:** Older adult, Rehabilitation, Post-acute care, Care transitions, Prediction, Innovative care model

## Abstract

**Background:**

Understanding the provision of health services to community-dwelling older adults is of great importance due to regulatory changes within post-acute care. The aim of this study was to illustrate pathways by which older adults, within an innovative post-acute care delivery model, move to either independence or re-admission back into higher levels of care to maximize the value of rehabilitation delivery.

**Methods:**

Clinical data specific to an episode of care (*n* = 30,001) provided to Medicare beneficiaries treated via a rehabilitation house-calls model of care in their homes and senior living communites were separated into training and test sets. Classification trees were fit on the training set’s administrative and clinical variables. Descriptive statistics were calculated for the overall sample, patient characteristics, clinical characteristics, and clinical outcomes.

**Results:**

Subjects were 83.3 years on average, 69.4% were female, and 62.2% were seen in their own homes while 37.8% were in senior living. The key variables predictive of progressing to independence were total number of visits, the presence of the Patient Specific Functional Scale (PSFS), PSFS score at discharge and change in PSFS. Prediction accuracy of the classification tree on the test set was 82.4%.

**Conclusions:**

Older adults progress to a higher degree of independence, instead of higher levels of care, via several distinct pathways within a rehabilitation house-calls model of care. A mix of service utilization and outcome variables are key predictors of each pathway and may be used to maximize the value of service delivery. Further examination of the predictors of outcome using administrative datasets drawn from different sub-sets of older adults across the post-acute care continuum is warranted.

## Background

Value-based healthcare is defined as a system where providers are paid based on patient outcomes. A rapidly aging United States population is magnifying concern over value in healthcare. Many countries including Germany, Italy, France, Spain, and Japan have been experiencing aging populations for some time, but are no better aligned with value-based healthcare than the US [1].

Medicare Part A pays for inpatient care, skilled nursing facilities (SNF), hospice, and home health care while Medicare Part B pays for outpatient services. The Medicare Payment Advisory Commission (MedPac), an agency that provides non-partisan analysis of the United States (US) Medicare program, has long expressed concern that Medicare Part A home health agencies (HHA) “target therapy visit thresholds used to adjust payment…targeting the ranges that appear most profitable.” [9].

The Centers for Medicare & Medicaid Services (CMS), the agency that oversees the Medicare system, introduced the Patient-Driven Groupings Model (PDGM) which will change home health payment in two primary ways beginning in 2020. First, therapy will be removed as a determinant of payment. Second, episodes of care will be reduced from 60 days to 30 days. The progression of value-based legislation, including the PDGM, will likely limit the provision of home health therapy under Part A which is where most rehabilitation services are provided to older adults within the post-acute continuum [11]. As a result, the healthcare system will see an increase in frail, and potentially costly, older adults without home therapy services whose functional incapacitation may limit access to traditional outpatient settings.

Healthcare administrators will need to think beyond a post-acute model of traditional clinic-based outpatient physical therapy services and consider alternative delivery models that enhances access for older adults. In addition, the demand to match the model of service delivery and the outcomes of care will be greater than ever before. “Rehabilitation house-calls” is an innovative model of post-acute rehabilitation that provides geriatric specific outpatient services in the patient’s residence, similar to home health, but under the Medicare Part B outpatient benefit.

Our current knowledge is predicated on studies that have typically sampled only older adults receiving traditional Part A home health or Part B outpatient rehabilitation. Therefore, we know little about the outcomes of rehabilitation of these relatively immobile older adults who are inappropriate for Part A services and may not have reached their full functional potential. This sub-population of older adults will likely grow due to impending legislation and are not well understood as few data sets currently capture utilization and rehabilitation outcomes that promote independence and reduce re-admission back into higher levels of care. This unique model of care provided an opportunity to explore these issues.

Therefore, the aim of this study was to illustrate pathways by which older adults receiving rehabilitation house calls might move to either independence or re-admission back into higher levels of care to influence the value of this innovative care delivery model inside and outside of the US.

## Methods

### Study design

Retrospective review of an administrative database.

### Data source

A de-identified dataset was available for the study with 38,203 physical therapy episodes of care provided to 30,001 Medicare beneficiaries between October 31st, 2014 and September 30th, 2016 within a single private practice. The dataset included patient ages in five-year increments. However, to ensure proper de-identification, ages of at least 90 years were aggregated into a single group. The first observed episode of care for each unique beneficiary within the 2 year time period was extracted to produce an analysis subset of 30,001 episodes of care. This study was reviewed and exempted as human subjects research by a research ethics board.

Data were collected during care delivered to community-dwelling older adults via a rehabilitation house-calls model of care within their own homes or a senior living community. Clinical teams are organized regionally with operational and clinical leadership supported centrally. Data for this study were culled from the practice-wide electronic health record (EHR) from documentation entered by both salaried and per-diem physical therapists. All data were captured during normal interactions between clinicians and their patients.

Most data values were direct extractions, other data, e.g. number of treating physical therapists, used in this study were aggregated from the data in the EHR. The PSFS is a measure of physical function that is reliable and valid in community-dwelling older adults [8] that was a direct extraction from the EHR. The patient’s ability to complete selected activities is rated using an 11-point scale. Therefore, the assessment has large applicability and utility within the older adult population as only 25.3% of our sample did not have a PSFS recorded at evaluation and discharge. All patient characteristics were taken from data recorded during the initial evaluation process, while the details regarding clinical care were extracted using the entire plan of care.

### Construction of rehabilitation outcomes

The nine discharge reasons are structured fields located on the discharge note within the EHR that indicate the status of the patient at the time of discharge. Eight of the nine reasons are forced-response options directly extracted from the system. Responses in the “other” discharge reason category are free-text and a Delphi survey was utilized to operationalize those cases into a more defined discharge reason [12]. Only 14.1% of the data required interpretation using the Delphi rubric.

Five reviewers with varying responsibilities within the practice, e.g., documentation review, regional operations, and quality assurance were provided a table of potential operational definitions from the “other” category and asked to associate each with a discharge reason and each discharge reason with a construct. All responses were returned directly to the authors and structured summary responses for further review were sent to each participant individually. If three of the five reviewers believed that a definition belonged within a different reason, it was moved. If no more than two reviewers agreed a definition belonged within a reason, it was removed. If the reviewers made no comment, the reason for discharge was retained in its initial discharge reason.

After the initial Delphi round it became clear that the response options alone were not sufficient to assign each discharge reason to a positive, negative or ambiguous construct. Therefore, the discharge reason of no further skill required was determined to be a positive outcome while all other forced discharge reasons were grouped as outcomes that were poor or potentially outside the scope of physical therapy. The poor outcomes were grouped because the administrative records could not conclusively distinguish between outcomes that resulted from insufficient care and those caused by the myriad factors which may contribute to such outcomes that are outside the scope of physical therapy.

The feedback from the Delphi survey was integrated into a second Delphi round and presented to all reviewers. The second Delphi survey did not identify any further changes and the operationalization of all definitions relevant to this study by the Delphi survey was determined to have appropriate face validity by the authors and practice reviewers. The discharge reasons, operational definitions and assigned constructs are given in Table 1.

**Table 1:** Discharge reasons, operational definitions and construct assignment

Discharge Reasons	Operational Definitions	Construct Assignment^a^
Patient does not require skilled therapy • 383 identified via Delphi	1. Transition to home exercise program (HEP), exercise physiologist or private pay services 2. Met/Achieved goal(s) 3. Demonstrating independence with HEP 4. Max potential with skilled services 5. Functional plateau 6. Achieved prior level of function 7. The patient was referred for a wheelchair assessment only and does not require skilled services until the wheelchair is received	Positive
Patient declined services • 621 identified via Delphi	1. Death in family 2. Refusal 3. Patient choice 4. Insurance coverage 5. Financial constraints 6. Self discharge (DC) because they felt they met goals 7. Self DC	Poor, but potentially outside the scope of physical therapy
Patient hospitalized • 64 identified via Delphi		Poor, but potentially outside the scope of physical therapy
Patient transitioned to home health or hospice services • 313 identified via Delphi	1. Needs “nursing”, but not admitted to SNF 2. “Med A”	Poor, but potentially outside the scope of physical therapy
Other	1. Transition to other discipline with no explanation of why current plan of care (POC) ended 2. Decline in function and/or cognition 3. “See assessment” 4. Coverage issue 5. Admitted to senior living community (SLC) 6. Facility changing provider 7. Physician DC 8. Medical hold 9. Returned home 10. Change in medical status	Categorized based on Delphi results

^a^The following discharge reasons were not analyzed since they are outside the scope of physical therapy or highly infrequent within the sample: not adherent to plan of care, patient expired, unable to obtain consent to care, patient sent to sub-acute rehab or SNF

### Data analysis

A classification tree was developed to stratify the population of interest into proportions of individuals whose personal and treatment characteristics are associated with high or low rates of achieving the desired outcome (i.e. patient no longer required skilled therapy). Unlike other regression approaches, classification trees accomplish this by recursively partitioning the sample to uncovering interactions between and among independent variables that occur only for segments of the entire sample [6]. Consequently, classification trees are able to identify important predictors for specific at risk proportions of individuals even when those predictors may not be meaningful for the population as a whole.

Pathways within the tree in this manuscript represent patient-specific predictions of eventual independence from therapy based on simple criteria. The classification tree is determined by an algorithm that considers every value of every variable to determine the splits necessary to maximize the homogeneity of the two resulting nodes. That is, so that patients on one side of a split are significantly more likely to achieve the desired outcome while those on the other side of the split are less likely.

The classification tree reported below was identified through a multi-stage process as follows. A random forest of 500 classification trees to predict desired outcome was produced using all variables with at least 10,000 observations as a dimension-reduction technique to remove weakly explanatory variables. Variables with high levels of missing values were not included in the random forest as random forests, unlike classification trees, can only use subjects with complete data. Indicator variables for presence of clinical test data were created and used in the random forest. This was done to account for high rates of missing data in these variables in the event their presence was informative. Variables in the random forest were then ranked by their mean Gini increase, a measure of how much node homogeneity is contributed by a variable, and the worst-performing 15% of variables were dropped from subsequent classification tree analyses. The mean Gini increase for each variable is given in Table 2.

**Table 2:** Mean gini increase from the random forest

Variable	Mean Gini Increase	Dropped
Age	346.696	
Sex	52.953	
Place of service	51.300	
State	424.932	
Chronic conditions		
Parkinson’s disease (PD)	34.686	
Congestive heart failure (CHF)	31.510	
Chronic obstructive pulmonary disease (COPD)	25.037	
Cerebrovasular accident (CVA)	35.441	
Diabetes mellitus	42.755	
Total hip arthroplasty (THA)	23.602	
Total knee arthroplasty (TKA)	29.201	
Dementia	59.046	
Depression	41.987	
Visits	458.771	
Optimal Living (OL) Resident	14.441	
Current procedural terminology (CPT) billing code percentage		
97110	426.949	
97530	436.353	
97116	423.157	
97112	366.139	
97001	484.137	
97140	187.353	
97750	227.107	
97535	130.800	
97542	42.704	
97124	51.238	
97035	23.398	
97032	9.585	Yes
97002	28.437	
97113	16.674	
97760	7.566	Yes
97532	10.698	
95992	68.344	
97761	1.656	Yes
97537	2.346	Yes
97762	2.811	Yes
97533	0.911	Yes
97034	1.182	Yes
97012	0.225	Yes
97018	0.0	Yes
97597	0.0	Yes
97033	0.047	Yes
97602	0.0	Yes
97598	0.0	Yes
Number of treating physical therapists	77.706	
Functional Outcome Measures (FOMs)		
Timed up and go (TUG)	62.481	
Dual task TUG	5.476	Yes
Gait speed	7.620	Yes
Five time sit to stand	59.018	
Berg balance scale	37.144	
Functional reach	33.512	
Six minute walk test	28.284	
PSFS at evaluation	423.061	
PSFS at discharge	1687.624	
Change in PSFS (discharge – evaluation)	1148.959	
Visits per week	534.986	
Total treatment minutes	534.074	
Delay in care	313.766	
Admission to assignment	170.976	
Assignment to examination	298.181	
Units per visit	393.090	
Units per episode	484.598	
Cost per episode	568.501	
Concurrent care visits OT	221.423	
Concurrent care visits SLP	81.872	
Concurrent care visits EP	1.700	Yes

Before fitting the classification tree, the dataset was randomized into an 80% training set and 20% test set for purposes of cross-validation. A collection of classification trees was fit on the training set by varying the complexity parameter between 0.002 and 0.01. This parameter determines the smallest increase in prediction accuracy necessary for a split to exist in the tree. Its purpose is to reduce model overfitting. The final classification tree was then determined by the standard practice of setting the complexity parameter to be the largest possible such that the corresponding tree’s cross-validated error was no more than the minimum cross-validated error plus 1 standard deviation of that error [4]. The predictive accuracy of this tree was determined using the 20% test set.

All statistical analyses were performed using R version 3.3.2 [13]. Random forests and classification trees were built using the random Forest and rpart packages in R [7, 14]. Statistical significance was set at *p* < 0.05.

## Results

A total of 30,001 episodes of care occurring from October 31st 2014 to September 30th 2016 were analyzed. Patients had a mean age of 83.3 years and 20,757 (69.4%) were female. Patients were seen by a single clinician for the duration of their episode 85% (25,511) of the time, by two clinicians 12.2% (3651) of the time, and by 3 or more clinicians in 2.8% (841) of episodes. Medicare was the primary payer on all episodes included in this study. Additional, patient characteristics are given in Table 3, clinical characteristics in Table 4, and rehabilitation outcome results in Table 5. Nine chronic conditions, particularly common in older adults, had incidence rates ranging from 4.9 to 28.1%, with dementia (28.1%), diabetes mellitus (17.6%), and depression (17%) being most prevalent in the sample. Overall, 56% of the sample achieved the desired outcome of independence from therapy.

**Table 3:** Sample patient characteristics

Characteristic	Mean (SD) or N (%)
Age	
Under 60	943 (3.1)
60–64	580 (1.9)
65–69	1588 (5.3)
70–74	2400 (8.0)
75–79	3670 (12.2)
80–84	5906 (19.7)
85–89	7612 (25.4)
90+	7302 (24.3)
Sex: female	20,757 (69.4)
Place of service	
Community	19,439 (64.8)
Senior living community	10,562 (35.2)
Chronic conditions	
PD	2450 (8.1)
CHF	1998 (6.7)
COPD	1541 (5.1)
CVA	3006 (10.0)
Diabetes mellitus	5447 (18.2)
THA	1495 (5.0)
TKA	1947 (6.5)
Dementia	7966 (26.6)
Depression	4852 (16.2)

*Zero percent of variables were missing except for sex (0.003)

**Table 4:** Sample clinical characteristics

Characteristic	Mean (SD) or N (%)	Missing (%)
Visits	18.7 (14.3)	0 (0.0)
OL resident	638 (2.1)	0 (0.0)
CPT code percentage (5 most common)		0 (0.0)
97110	33.3 (17.6)	
97530	24.3 (15.8)	
97116	19.8 (13.2)	
97112	12.2 (13.1)	
97001	4.8 (8.6)	
Number of treating physical therapists	1.2 (0.5)	0 (0.0)
FOMs present at baseline and discharge		
TUG	35.5 (25.5)	18,389 (61.3)
Dual task TUG	6.3 (9.1)	29,906 (99.7)
Gait speed	0.53 (0.22)	29,804 (99.3)
Five time sit to stand	32.8 (19.6)	23,708 (61.6)
30 s sit to stand	4.1 (2.8)	27,449 (91.5)
Berg balance scale	32.2 (11.0)	26,217 (87.4)
Functional reach	4.6 (2.3)	27,919 (93.1)
Six minute walk test	129.3 (90.2)	27,921 (93.1)
PSFS	3.5 (1.9)	7601 (25.3)

**Table 5:** Sample rehabilitation outcome results

Discharge Reasons	Frequency	Percent of Sample
Patient no longer requires skilled therapy	16,668	55.6%
Patient declined services	3783	12.6%
Patient hospitalized	2959	9.9%
Patient transitioned to home health or hospice services	1756	5.9%
Not adherent to POC	874	2.9%
Patient expired	400	1.3%
Unable to obtain consent to care	27	0.1%
Patient sent to sub-acute rehab or SNF	0	0%
Other	4238	14.1%

The mean Gini coefficient criterion used with the random forest resulted in indicators for dual task TUG and gait speed being dropped from consideration, as well as the number of concurrent EP visits, and eight payment codes that combined for less than 0.1% of billing.

**Fig. 1:**
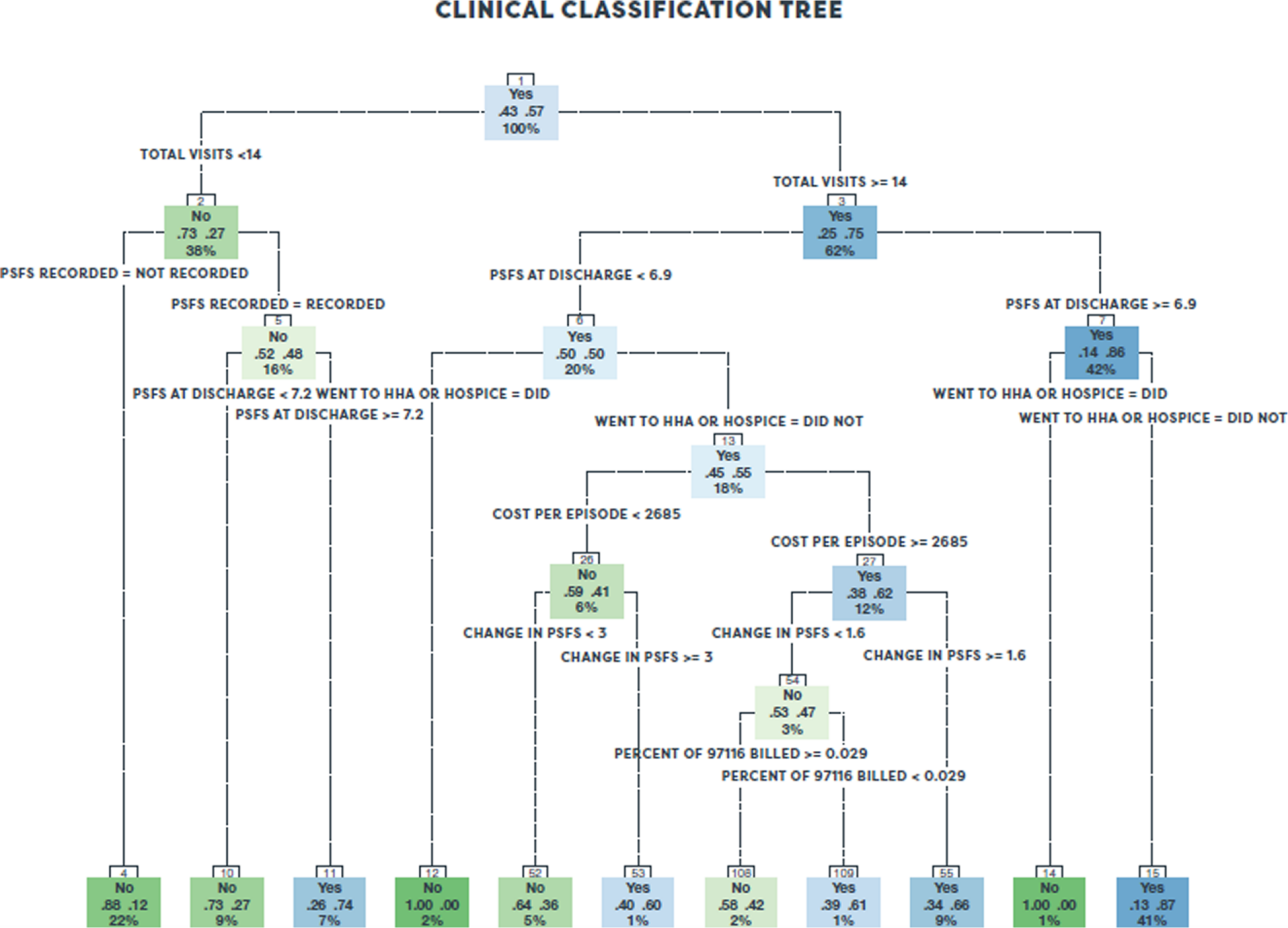
Clinical classification tree

Classification trees were fit on all variables not excluded by the random forest to determine characteristics and cutoffs that predicted a patient progressing to independence from therapy. The results are displayed within Fig. 1. Tree nodes are green if the majority of patients in that node did not achieve a desired outcome and blue otherwise. The darkness of the node indicates the overall percentage of achievement or non-achievement. Predicted outcomes are determined by the majority result at each terminal node of the classification tree.

The classification tree divides the sample into four primary proportions of individuals among those that did not go to home health or hospice care. The high care, high achievement group is comprised of those who received at least 14 visits and had a PSFS at discharge of at least 6.9. This group represented 41% of the total sample, of which 87% of them had desired outcomes. The low care, low achievement group is comprised of those with fewer than 14 visits, and either their PSFS at discharge was below 7.2 or they never had a recorded PSFS. This group, represented by terminal nodes 4 and 10 in Fig. 1, represents 31% of the total sample, of which 16.4% of this group had desired outcomes. There was a low care, high achievement group that received fewer than 14 visits but achieved a PSFS of at least 7.2 at discharge. This group was 7% of the sample, of which 74% of them achieved a desired outcome. Finally, there was a high care, indeterminate achievement group that received at least 14 visits but had a PSFS at discharge of less than 6.9. This group was 18% of the sample, of which 55% had desired outcomes.

Due to the predictive nature of the presence of PSFS scores, we conducted post-hoc comparisons of baseline characteristics between the 22,400 patients with PSFS scores and the 7601 without. The PSFS subgroup had higher rates of PD, COPD, and Depression (*p* ≦ 0.002), and were more likely to be female (*p* = 0.026). Differences between the age range groups were detected by chi-square test (*p* < 0.001), where the no-PSFS group had a somewhat higher proportion of under-65 s and fewer in the 85+ age groups. No significant differences were found in service location, OL residency, or the other chronic conditions of interest. The differences between groups support the applicability of the PSFS as a measure for older adults with common chronic diseases as well as the clinical decision to forgo the PSFS within the no-PSFS group.

## Discussion

This study utilized an administrative data set from a large outpatient private practice to describe characteristics and variables predictive of progression to independence from therapy in Medicare beneficiaries receiving care via a “rehabilitation house-calls” model. This model of care was designed to serve a subset of the older adult population that is frail, potentially high cost due to re-admission risk, and has not been well represented in the literature, but will also grow due to value-based regulations within the post-acute continuum. Inclusion and exclusion criteria for this study were purposely kept minimal so that the results would provide insight into resource utilization within this model of care across all patient clusters: individuals who clearly benefit from therapy, those that may benefit depending on the situation, and those that are likely to experience limited functional value from rehabilitation intervention.

Clinical outcomes were best predicted in the classification tree by a mix of service utilization variables and the PSFS, which is a questionnaire used to quantify gross functional improvement or ability. We found that 63% of patients fell into two highly predictive terminal nodes (4 and 15) determined entirely by their number of visits and PSFS score. Among those with fewer than 14 visits who had no PSFS recorded, 88% failed to achieve a desired outcome, while among those individuals with at least 14 total visits who had a PSFS of 6.9 or higher at discharge, 87% achieved a desired outcome.

Although our intention was to fit a model that would predict progression to independence from therapy, our findings also provide insight into factors that influence the risk of re-admission back into higher levels of care including the hospital, home health, and hospice. Terminal nodes with lower proportions of individuals who achieved the desired outcome identified particular groups at higher risk of hospitalization or going to home health or hospice (i.e. nodes 4, 10 and 52). In addition, an entire proportion of individuals with at least 14 visits that did not achieve a discharge PSFS of at least 6.9 transitioned to home health or hospice (i.e. node 14) did not achieve the desired outcome. Based on the complementarity of these findings, we suggest that a discharge PSFS of at least 6.9 after at least 14 visits may be a meaningful threshold in predicting transition to home health or hospice that should subsequently be confirmed with additional data.

The older adults receiving services in this model are different than those accessing typical outpatient services. The sample population used by Fritz et al. [2] in 2011 is an apt comparison since they analyzed Medicare beneficiaries over a 2 year period using an equivalent definition of episode of care. Demographically, the populations differed in age by an average of 9.1 years (83.3 vs 74.2) and gender by 4.1% (69.4 vs 65.3) with more of this sample population being older and female. The vast majority of the Fritz population (99.1%) presented with musculoskeletal conditions as the primary diagnoses while much of our sample presented with multiple chronic conditions and many were hospitalized prior to beginning therapy. These key differences limit our ability to benchmark the outcomes of this unique care delivery model, but support the utility of this model as an alternative to home health or terminal end of the post-acute continuum for older adults. Using pain as the outcome, Fritz et al. concluded that better outcomes occurred when there was greater initial disability (pain), and more utilization. Our findings are consistent with Fritz as those who received more visits (> 14) were more likely to achieve the desired outcome, and progress made during the episode of care and discharge functional level were predictive within this particular model.

We also found that there was a small group of subjects who had at least 20 rehabilitation house-call visits and achieved the desired outcome at a significantly higher rate. Upon further examination, it seems that these individuals were in a private pay wellness continuum program called Optimal Living (OL) within their senior living communities.. Care coordination for frail older adults is lacking within and outside the US [3, 10] and several studies have demonstrated reduced healthcare costs and hospitalizations when care is better coordinated [5, 10]. The OL program includes systematic resident monitoring, exercise classes that are specific to a resident’s performance on selected screening assessments, and care coordination with appropriate transition between skilled rehabilitative or maintenance care and wellness programming. At this time we are unable to identify the “active ingredient” in OL, but it appears that a full time rehab director trained to care for older adults, properly dosed wellness programming, care coordination, and surveillance by a therapy team working closely with the older adults may be a potent model of service within long-term care that significantly impacts functional status [10].

The size, scope, and structure of this administrative dataset were beneficial from a statistical perspective, but several limitations should be considered. First, this study included data from a single provider practicing in a certain geographic location across the United States over a two-year period, and was limited in the variables that were available to be studied. Other factors, which might also be able to predict the outcome such as education level and inpatient hospitalization diagnosis prior to admission, were not available in the dataset.

The dataset was extracted from a patient service record rather than prospectively using a research protocol which necessitated the use of a Delphi survey. Although anonymous to each other, the Delphi panelists were known to the authors to ensure representation from documentation review, regional operations, and quality assurance. The operational definition of “functional plateau” was placed under the discharge reason of “patient doesn’t require skilled care” because the teaching, training, documentation review, and auditing systems within this particular private practice are such that the likelihood a patient is discharged due to “functional plateau” without the consideration of rehabilitation approach and mitigation of progress due to decline is minimal. All clinicians are within a single practice with the same training, regional quality support and auditing. Therefore, we assumed a high degree of similarity in terminology within those entering the data and did not validate our approach.

Many of the functional outcome measures had high rates of missing data. Some variables were dependent on patient self-report and clinician input which might be a source of bias. The episode of care in the study was usually, but not always, the initial episode of care in the study’s timeframe. Specifically, the initial episode was the first for 20,326 patients and the second episode for an additional 5154 patients. Among the remaining 4521, 75% were on their third or fourth episode while the remainder was between their fifth and thirteenth episode. A definition of “undesirable” to complement “desirable” was not able to be constructed because many of the undesirable outcomes could not be clearly associated with deficiencies in care or as exacerbated by care, particularly given the advanced age of the sample. Lastly, data on interventions was derived from CPT codes which broadly define interventions for billing purposes and therefore diminishes the ability to account for exact details of treatment.

## Conclusion

Using classification trees developed from a sample of 30,001 Medicare beneficiaries, we identified several distinct pathways by which patients progressed to a higher degree of independence rather than higher levels of care. This innovative model of post-acute care should be emphasized to enhance access as post-acute care regulations evolve. In addition, the sample is unique and not well represented within current literature. These pathways provide insight into functional outcomes and resource utilization that may be used to maximize the value of service delivery within post-acute care.

## Abbreviations

CHF: Congestive heart failure
CMS: Centers for Medicare & Medicaid Services
COPD: Chronic obstructive pulmonary disease
CPT: Current procedural terminology
CVA: Cerebrovascular accident
DC: Discharge
EHR: Electronic health record
FOM: Functional outcome measure
HEP: Home exercise program
HHA: Home health agency
MedPac: Medicare Payment Advisory Commission
OL: Optimal Living
PD: Parkinson’s disease
PDGM: Patient-Driven Groupings Model
POC: Plan of care
PSFS: Patient Specific Functional Scale
SLC: Senior living community
SNF: Skilled nursing facility
THA: Total hip arthroplasty
TKA: Total knee arthroplasty
TUG: Timed up and go
US: United States

## References

[1] Agrawal T. Value-based healthcare: a global report. The Economist Intelligence Unit. 2016; Available from: http://vbhcglobalassessment.eiu.com/value-based-healthcare-a-global-assessment/. Accessed 26 Mar 2019.

[2] Fritz JM, Hunter SJ, Tracy DM, Brennan GP (2011). Utilization and clinical outcomes of outpatient physical therapy for Medicare beneficiaries with musculoskeletal conditions. Phys Ther.

[3] Hansson A, Svensson A, Ahlström BH, Larsson LG, Forsman B, Alsén P (2017). Flawed communications: health professionals’ experience of collaboration in the care of frail elderly patients. Scandinavian Journal of Public Health.

[4] Hastie T, Tibshirani R, Friedman JH (2009). The elements of statistical learning: data mining, inference, and prediction.

[5] Kim Y-E, Hong S-W (2018). Health-related effects of the elderly care program. Biomed Res Int.

[6] Lemon SC, Roy J, Clark MA, Friedmann PD, Rakowski W (2003). Classification and regression tree analysis in public health: methodological review and comparison with logistic regression. Ann Behav Med.

[7] Liaw A, Wiener M (2002). Classification and regression by randomForest. Rnews..

[8] Mathis RA, Taylor JD, Odom BH, Lairamore C. Reliability and validity of the patient-specific functional scale in community-dwelling older adults. J Geriatr Phys Ther. 2018; Available from: 10.1519/JPT.0000000000000188. 10.1519/JPT.0000000000000188 29630006

[9] MedPac. Report to the Congress: Medicare Payment Policy. 2011 Mar p. 182–3.

[10] Mukamel D, Peterson D, Temkin-Greener H, Delavan R, Gross D, Kunitz SJ (2007). Program characteristics and enrollees’ outcomes in the program of all-inclusive Care for the Elderly (PACE). The Milbank Quarterly.

[11] National Center for Health Statistics (U.S.), editor. Long-term care services in the United States: 2013 overview. Hyattsville, Maryland: U.S. Department of Health and Human Services, Centers for Disease Control and Prevention, National Center for Health Statistics; 2015. (Vital and health statistics. Series 3, Analytical and epidemiological studies). 26158640

[12] Portney L, Watkins M (2015). Foundations of clinical research.

[13] R Core Team. R: A language and environment for statistical computing. R Foundation for Statistical Computing. Vienna, Austria; 2017. Available from: https://www.R-project.org/. Accessed 3 Aug 2017.

[14] Therneau T, Atkinson B (2018). Rpart: recursive partitioning and regression trees.

